# Supplementary material for: A closer look at weight loss interventions in primary care: a systematic review and meta-analysis
Source: Front Med (Lausanne). 2023 Nov 23;10:1204849. doi: 10.3389/fmed.2023.1204849 (PMC10701393; doi:10.3389/fmed.2023.1204849)
Supplement: Supplementary file 1 [file Data_Sheet_1.docx]

**Supplement. Data and code for the meta-analysis**

library(meta)

library(metafor)

library(xlsx)

rm(list=ls())

data<-read.xlsx("data.xlsx",sheetIndex = 1)

data$N[data$ITT!="ITT"]<-data$NAtOutcome[data$ITT!="ITT"]

noncontrol<-subset(data,Arm!="Cntrl")

control<-subset(data,Arm=="Cntrl")

## intervention = X, control = Y

composite<-merge(noncontrol,control,by=c("Author","Year","FU"))

composite<-transform(composite,Study=paste(Author,Year,Arm.x),AUYR=paste(Author,Year))

composite<-composite[order(composite$FU,composite$Year),]

composite<-transform(composite,FU = paste(FU,"months"),Time=FU)

## synthesize studies with multiple reported TE

fAUb<-metacont(n.e=N.x,mean.e=TE.x,sd.e=TE.se.x,

n.c=N.y,mean.c=TE.y,sd.c=TE.se.y,

studlab=AUYR,data=composite,prediction=F,byvar=AUYR,

method.tau="REML",bylab="Study",comb.fixed=FALSE,

subset=!is.element(Author,c("Lindgarde et al","Martin et al","Boesten et al")))

## get rid of labels for the plot

fAUb$studlab<-rep("",length(fAUb$studlab))

png("forestbyAuthor.png",width=8,height=6,units="in",res=600,pointsize=10)

forest(fAUb,leftcols = c("studlab" ,"Arm.x","FU","n.e",

"mean.e", "sd.e", "n.c", "mean.c", "sd.c"),

leftlabs = c("Intervention","Follow-up"),digits=2,digits.se = 2,

smlab="Control Corrected\nWeight Loss (kg)",fontsize=7,overall=FALSE)

dev.off()

## funnel plot ;

## aggregate studies and then do the funnel plot

fAU<-metacont(n.e=N.x,mean.e=TE.x,sd.e=TE.se.x,

n.c=N.y,mean.c=TE.y,sd.c=TE.se.y,

studlab=AUYR,data=composite,prediction=F,byvar=AUYR,

method.tau="REML",bylab="Study",comb.fixed=FALSE)

deriveddf<-data.frame(TE=fAU$TE.random.w,seTE=fAU$seTE.random.w,Study=fAU$bylevs)

## sort by year

deriveddf<-transform(deriveddf,Year=substr(Study,nchar(Study)-4,nchar(Study)))

deriveddf<-deriveddf[order(deriveddf$Year),]

ffunel<-metagen(TE=TE,seTE=seTE,data=deriveddf,studlab = Study,method.tau="REML")

png("funnel.png",width=6,height=6,res=600,pointsize=10,units="in")

fun<-funnel(ffunel, random = TRUE,

level = 0.95, contour = c(0.9, 0.95, 0.99),

col.contour = c("grey90", "grey80", "grey70") ,

lwd = 1, cex = 1, pch = 16, studlab = TRUE, cex.studlab = 0.6,

xlim=c(-11,4),pos.studlab=c(3,3,4,3,4,1,2),xlab="Mean Difference in Weight (kg)")

legend(-10, 0.05,title="Study p-value",

legend=c("0.1 > p > 0.05", "0.05 > p > 0.01", "p< 0.01"),

fill = c("grey90", "grey80", "grey70"))

dev.off()

## egger test

regtest(x=deriveddf$TE,sei=deriveddf$seTE, model="rma")

## meta-analysis

png("meta-analysis.png",width=6.5,height=4,units="in",res=600)

forest(ffunel,prediction=T,smlab="Control Corrected\nWeight Loss (kg)",

digits=2,digits.se = 2,fontsize=7)

dev.off()

##metaregression with the package metafor

## prepare the data first

prepESC<-escalc(measure="MD",m1i=TE.x,sd1i=TE.se.x,n1i=N.x,

m2i=TE.y,sd2i=TE.se.y,n2i=N.y,data=composite,slab=AUYR)

## random effect at the study level

TimeEffect<-rma.mv(yi,vi,data=prepESC, random = ~ 1 | AUYR/FU,mods=~I(Time/6))

DrugEffect<-rma.mv(yi,vi,data=prepESC, random = ~ 1 | AUYR/FU,mods=~Intervention.x!="Non-drug")

TimeDrugEffect<-rma.mv(yi,vi,data=prepESC, random = ~ 1 | AUYR/FU,mods=~I(Intervention.x!="Non-drug")+I(Time/6))

TimeDrugEffectInteract<-rma.mv(yi,vi,data=prepESC, random = ~ 1 | AUYR/FU,mods=~I(Intervention.x!="Non-drug")*I(Time/6))
